# Encampment sweeps and persistent homelessness among unhoused people who use drugs in Vancouver, Canada: A prospective cohort study

**DOI:** 10.1371/journal.pmed.1005033

**Published:** 2026-09-24

**Authors:** Kanna Hayashi, Eric C. Sayre, Jennifer McDermid, Samona Marsh, Caitlin Shane, Michael-John Sheridan Milloy, P.O.W.E.R., Thomas Kerr

**Affiliations:** 1 British Columbia Centre on Substance Use, Vancouver, British Columbia, Canada; 2 Faculty of Health Sciences, Simon Fraser University, Burnaby, British Columbia, Canada; 3 Police Oversight With Evidence and Research, Vancouver, British Columbia, Canada; 4 Interdisciplinary Studies Graduate Program, University of British Columbia, Vancouver, British Columbia, Canada; 5 Vancouver Area Network of Drug Users, Vancouver, British Columbia, Canada; 6 Coalition of Peers Dismantling the Drug War, Vancouver, British Columbia, Canada; 7 Research 101 and a Manifesto for Ethical Research in the Downtown Eastside, DTES Community Research Ethics Workshop (CREW), University of British Columbia Learning Exchange, Vancouver, British Columbia, Canada; 8 Pivot Legal Society, Vancouver, British Columbia, Canada; 9 Department of Medicine, University of British Columbia, Vancouver, British Columbia, Canada; United Nations Relief and Works Agency for Palestine Refugees in the Near East Jordan: UNRWA Jordan, JORDAN

## Abstract

**Background:**

Housing is a key determinant of health. In response to increasingly visible homelessness amid a growing housing crisis, many jurisdictions in North America forcibly displace unhoused people from public spaces, routinely confiscating their personal belongings. While prior research demonstrated various health-related harms associated with these “encampment sweeps,” the impact on housing placements among otherwise unhoused people remains unclear. We sought to estimate the relationship between experiencing encampment sweeps and transitioning from homelessness among unhoused people who use drugs in Vancouver, Canada.

**Methods and findings:**

Data were derived from two harmonized prospective cohort studies of people who use drugs in Vancouver, followed through semi-annual interviews between December 1, 2021 and May 31, 2025. We used proportional hazard marginal structural modeling (MSM) to examine if experiencing municipal government-enforced confiscation of personal belongings was associated with no longer reporting homelessness in the past 6 months during follow-up among unhoused participants after adjusting for a range of potential time-varying confounders including demographic characteristics, drug use patterns, health status and other social/structural exposures. A secondary analysis replaced confiscation of belongings with municipal government-enforced forced displacement that may have not involved confiscation of belongings between June 1, 2023 and May 31, 2025. Among 235 participants, 59 (25.1%) reported confiscation of their belongings at baseline. During follow-up, 128 (54.5%) stopped reporting homelessness, corresponding to an incidence rate of 55.6 (95% confidence interval [CI]: 46.4, 66.1) cases per 100 person-years. In the secondary analysis, 50 (32.5%) of 154 participants reported forced displacement at baseline and 62 (40.3%) stopped reporting homelessness during follow-up. In the proportional hazard MSM, confiscation of belongings (adjusted hazard ratio [AHR]: 0.35; 95% CI [0.13, 0.94]; *p* = 0.036) and forced displacement (AHR: 0.07; 95% CI [0.02, 0.20]; *p* < 0.001) were both negatively associated with transitioning from homelessness. Main limitations include potential overestimation of rates of transitioning from homelessness and limited causal inference due to the observational study design.

**Conclusions:**

The confiscation of personal belongings and forced displacement might undermine pathways to housing. Our findings underscore the need for policy approaches that prioritize access to dignified and affordable housing rather than practices that perpetuate homelessness. Future research should assess the quality of housing that unhoused people are placed in and examine how it relates to returning to homelessness.

## Author summary

**Why was this study done?:**

- Housing is a key determinant of health and many settings in North America have been facing a worsening housing crisis with an increasing number of people experiencing homelessness in recent years.
- In response, governments across North America have employed homeless encampment sweeps, in which unhoused people are forcibly displaced and have their belongings confiscated.
- While such encampment sweeps have been shown to have negative impacts on health and safety of unhoused people, the impacts on housing outcomes have not been clarified and we sought to address this research gap.

**What did the researchers do and find?:**

- In this study, we prospectively followed a community-recruited cohort of 235 unhoused people who use drugs in Vancouver, Canada.
- We asked our participants semi-annually about their experiences of encampment sweeps; whether they had their personal belongings confiscated by city workers between 2021 and 2025 and whether they were forcibly displaced by city workers between 2023 and 2025.
- We found that those who experienced municipal government-enforced confiscation of personal belongings or forced displacement were both less likely to stop reporting homelessness during study follow-up compared to other participants who did not experience encampment sweeps.

**What do these findings mean?:**

- The study findings indicate that encampment sweeps might undermine pathways to housing among unhoused people who use drugs in our study setting.
- Our findings underscore the need for policy approaches that prioritize access to dignified and affordable housing rather than practices that perpetuate homelessness.
- Key limitations of the current study include potential overestimation of rates of transitioning from homelessness to housing as our study might have tended to include unhoused people who were more connected with services; the quality of housing that participants were placed in was not captured; and limited causal inference.

## Abstract 3

In this prospective cohort study, Kanna Hayashi and colleagues examine whether encampment sweeps, including the confiscation of personal belongings and forced displacement, are associated with transitions out of homelessness among unhoused people who use drugs in Vancouver, Canada. Participants who experienced these interventions were less likely to stop reporting homelessness over time, highlighting concerns that such practices may hinder pathways to stable housing and underscoring the importance of housing-focused policy approaches.

## Introduction

Housing is a key determinant of health and is modifiable by public health and other policy interventions [1]. Despite such policy opportunity, regions across the United States (US) and Canada have faced a worsening housing crisis in recent years. While there is no universal definition of homelessness, the United Nations and other international and national organizations tend to conceptualize it along a continuum of situations characterized by a lack of stable, safe and adequate housing, ranging from living in public spaces or in places not intended for human habitation (i.e., unsheltered homelessness) to residing in emergency shelters, other temporary accommodations or housing that fails to meet public health and safety standards (i.e., sheltered homelessness) [2,3]. Between 2023 and 2024, the US had an 18% increase in the number of people experiencing homelessness (including sheltered and unsheltered) [4]. In the Greater Vancouver region in Canada, the number of individuals experiencing homelessness increased by 9% between 2023 and 2025 [5]. The city of Vancouver within this region experienced a slightly higher increase (12%) during this period, with 2,715 individuals identified as homeless in 2025 [5]. These numbers were generated using a point-in-time count method, which is known to result in undercounts of actual homelessness [5,6].

As a strategy to manage the growth of survival homeless encampments resulting from the housing crisis, many jurisdictions across the US and Canada increasingly forcibly displace unhoused people from public spaces [7–10]. Importantly, personal belongings, including those that are integral for survival (e.g., clothes, food, the structures of their homes, identification cards, and medications), are routinely confiscated during these processes [7,11,12]. Previous research has identified a number of severe health and safety harms associated with these practices (often called “forced/involuntary displacement” and “encampment/street sweeps”), including acute injuries, loss of survival items, intensified substance use and overdose, violent victimization, worsening mental health, and disconnection from health and social services [7,11–18]. As rates of substance use are known to be disproportionately high among unhoused people [19], a previous simulation modeling study involving 23 US cities over a 10-year period estimated that continual involuntary displacement might contribute to 16%–24% additional deaths among unhoused people who inject drugs [20]. Despite these immense public health harms, encampment sweeps continue to be endorsed and employed by multiple levels of government [21,22].

Previous qualitative research has indicated that encampment sweeps may undermine pathways to housing among unhoused people [8,9,11,13], yet few quantitative studies have investigated this issue at a population level. A previous audit report of the New York City (NYC) Comptroller documented that only 90 out of 2,308 unhoused people who were forcibly displaced by the NYC Department of Homeless Services in 2022 stayed in shelter for more than 1 day, and only three of them secured permanent housing thereafter [23]. However, this report also pointed to the inadequate tracking of placement referrals as being a significant limitation to their findings [23]. Therefore, we sought to leverage our longitudinal data generated from ongoing prospective cohort studies of community-recruited people who use drugs in Vancouver to examine if: (1) having one’s personal belongings confiscated; and (2) experiencing forced displacement; are associated with transitioning from homelessness during study follow-up among unhoused participants.

## Methods

### Ethics statement

The current study was approved by the Simon Fraser University Research Ethics Board (H24-02488). Written informed consent was obtained from all participants prior to be enrolled in the study.

### Study design, procedures, and participants

The reporting of this prospective cohort study aligns with the Strengthening the Reporting of Observational Studies in Epidemiology (STROBE) reporting guidelines (S1 Checklist). We did not use artificial intelligence tools. Self-reported longitudinal data were generated by two ongoing, harmonized open prospective cohort studies involving people who use drugs in Vancouver: the Vancouver Injection Drug Users Study (VIDUS) and the AIDS Care Cohort to evaluate Exposure to Survival Services (ACCESS). These cohorts have been described in previous publications [7]. In brief, during the current study period, VIDUS enrolled Human Immunodeficiency Virus (HIV)-seronegative adults (≥18 years of age) who injected drugs in the month before enrollment. On August 1, 2024, the eligibility criteria expanded to include at least weekly smoking of unregulated drugs in lieu of injection drug use. However, the number of smokers without a lifetime history of injection drug use is still low (i.e., two in the current study sample). ACCESS enrolled HIV-seropositive adults who used unregulated drugs in the month before enrollment. Other common eligibility criteria across the cohorts included residing in the Greater Vancouver region and providing written informed consent. Participants were recruited through word-of-mouth and street outreach primarily in the Downtown Eastside (DTES) neighborhood of Vancouver, an area characterized by high prevalence of marginalization and unregulated drug use as well as innovative community-led health interventions [24], including the development of encampment protocols and organized resistance to street sweeps [11]. Approximately half of unhoused people in the city of Vancouver reportedly reside in the DTES [10]. Data collection procedures were harmonized between the cohorts (e.g., administering the same questionnaire at the same frequency) to allow for pooled analyses. To maintain high follow-up rates, our frontline research staff obtained contact information for two persons who know the participant and conducted regular outreach to areas frequented by our participants. The staff includes those working with the cohort studies for more than 15 years and who have established rapport with our participants, which helped minimize some reporting bias, such as social desirability bias. Participants received an honorarium (the most recent amount during the current study period: Canadian $50) upon completion of each study visit.

At baseline and semi-annually thereafter, participants completed an interviewer-administered questionnaire which elicits a range of information including data on demographic data, substance use practices, healthcare access, and experiences with law enforcement. As survey questions related to municipal government-enforced confiscation of personal belongings were added to the questionnaire on December 1, 2021, for the primary analysis, sample eligibility criteria included: completing at least two interviews between December 1, 2021 and May 31, 2025, and reporting homelessness (self-defined and possibly including both sheltered and unsheltered homelessness) in the 6 month period prior to the first interview. For the secondary analysis focusing on municipal government-enforced forced displacement, which might or might not have involved confiscation of belongings, the study period was restricted to a period between June 1, 2023 and May 31, 2025, as the survey question about forced displacement was added on June 1, 2023.

### Measures

The primary outcome was no longer reporting homelessness in the past 6 months at any semi-annual follow-up interview during the study period, defined by answering “no” to the following question asked during follow-up “Have you been homeless in the last six months?” and reporting that they were living in some forms of housing other than temporary shelters at the time of the interview. Pre-listed response options in the questionnaire that were eligible for the outcome included: apartment/house (not including couch-surfing); modular housing; treatment/recovery house; and single-room occupancy hotels (SROs), which are small rental rooms with shared kitchen and bathroom facilities that are typically privately-owned, with many of them also serving as supportive housing, which the provincial government defines as long-term subsidized housing with on-site supports and services for low-income adults and families [10,25]. Participants were not provided legal definitions of these housing types. For those who reported a type of housing other than the pre-listed ones, we verified their free-form text responses for eligibility. Those who did not report homelessness in the past 6 months but reported currently having no fixed address or living on the street or in shelter at the time of the interviews were considered as remaining homeless and therefore continued to be followed in the time-to-event analyses, rather than being censored.

The primary explanatory variable of interest was experiencing municipal government-enforced confiscation of personal belongings (hereafter referred to as confiscation of belongings) in the past 6 months (yes *versus* no). As in our previous study [7], this variable was derived from a question: “Have you had your possessions confiscated by city workers in the last six months?” City workers included city engineering workers, park rangers and/or police officers. The secondary explanatory variable was experiencing municipal government-enforced forced displacement in the past 6 months (yes *versus* no), which was ascertained by asking the question: “In the last 6 months, have city workers or the police taken your tent (or other structures that you use as a place to sleep) away from public space (e.g., sidewalk)? Or did they try to take them away so that you had to pack up and leave?” Those who answered “yes” were subsequently asked a series of sub-questions including whether they were offered housing at the time of displacement, and whether they were able to successfully move in.

Based on the previous literature [7–9,11,13] and the lived experiences of some co-authors (i.e., some members of Police Oversight With Evidence and Research [P.O.W.E.R.], a community group advocating against violent law enforcement and largely comprised of people with lived expertise of substance use and housing instability; and the majority of whom are also members of the Vancouver Area Network of Drug Users and/or Western Aboriginal Harm Reduction Society), we considered a range of potential confounders. Demographic variables included: age (continuous, per year increase); self-identified gender (*cis-*woman, transgender or other *versus cis-*man); self-identified ethnicity/ancestry (white *versus* Indigenous or other persons of color); and current relationship status (legally married/common law/regular partner *versus* other). Health status-related variables included: having some or extreme barriers to mobility (*vs.* no barriers), as assessed by EuroQol 5-Dimension 3-Level (EQ-5D-3L), a validated survey instrument to measure quality of life [26]; and depressive and anxiety symptoms (moderate to severe *versus* mild to none), as assessed by the Patient-Reported Outcomes Measurement Information System (PROMIS) short forms 8b and 7a, respectively [27]. Substance use-related variables referring to the past 6 months included: ≥ daily use of unregulated opioids (i.e., heroin, fentanyl, or *down* [a colloquial term locally used to refer to unregulated opioids]), and ≥ daily use of unregulated stimulants (i.e., crystal methamphetamine or powder/crack cocaine). We also included DTES residence and informal or criminalized income generation activities (e.g., drug dealing, sex work, theft, etc.) in the past 6 months as well as self-reported lifetime experience of incarceration (as a time-updated variable) as key social and structural exposures. Unless otherwise stated, all variables were dichotomized as yes *versus* no and, except for ethnicity/ancestry that was ascertained once at the first study visit, treated as time-varying at each semi-annual follow-up cycle.

### Statistical analysis

We first examined the baseline sample characteristics stratified by experiencing confiscation of belongings, using the Kruskal–Wallis test (for age) and the exact chi-squared test (for all other variables). We generated a Kaplan–Meier curve for each sample for the analyses of the primary and secondary explanatory variables to visualize the probability of the event (no longer reporting homelessness) over time. While we did not collect data on the length of homelessness or the number of times participants experienced homelessness, among sub-samples of participants who completed a semi-annual interview prior to the baseline assessment for the current study, we counted the number of participants who reported incident homelessness in the past 6 months at baseline (i.e., not reporting homelessness in the past 6 months in a semi-annual interview preceding to the study baseline), the number and percentage of participants who experienced the event, and the median length of time to event.

**Fig 1:**
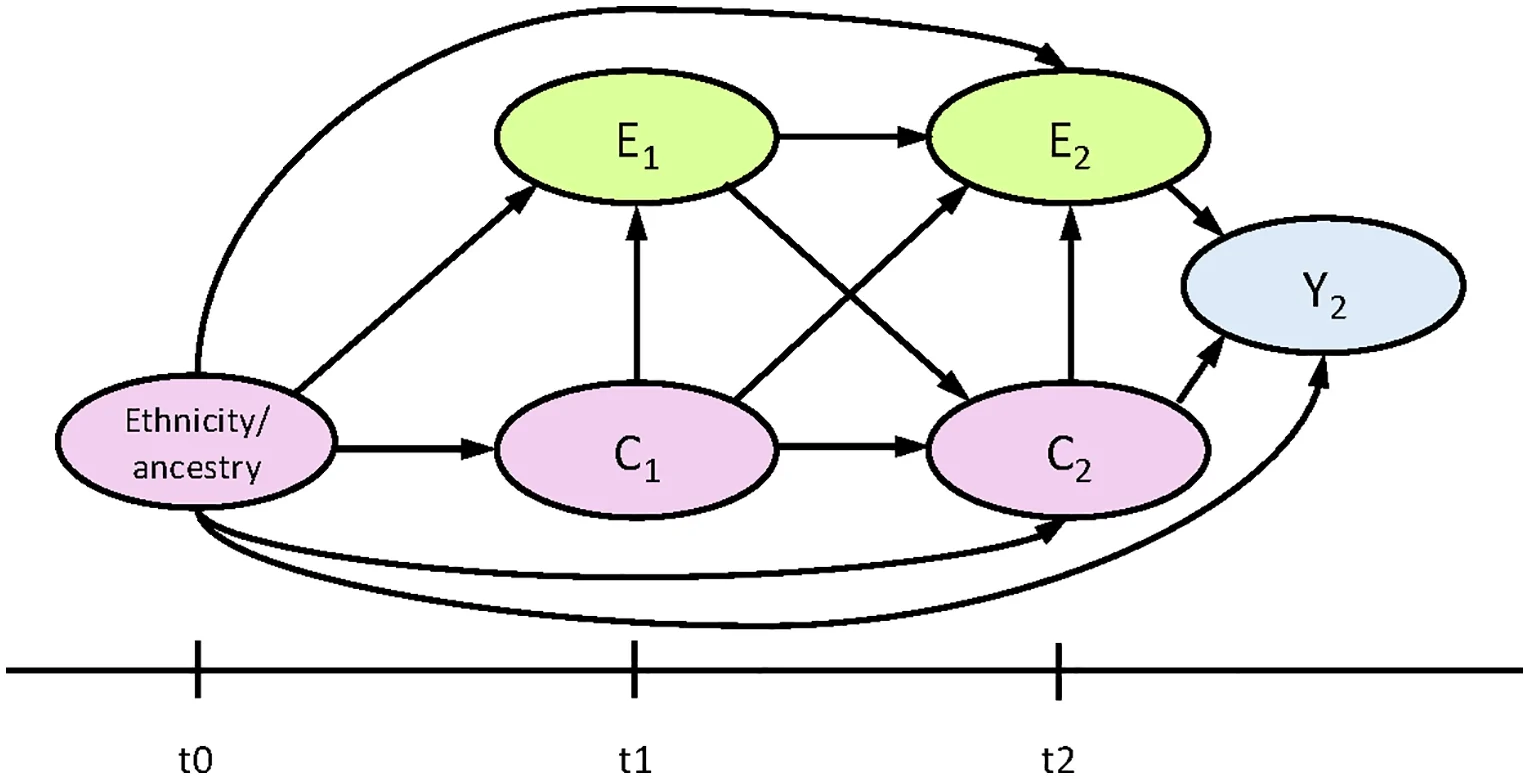
Directed acyclic graph depicting the hypothesized relationships among the exposure, the confounders, and the event. t0: Cohort enrollment; t1: First interview during the study period (baseline); t2: First follow-up interview during the study period; E: confiscation of belongings or forced displacement in the past 6 months; C: time-varying confounders; Y: no longer reporting homelessness in the past 6 months during study follow-up.

Next, for each of the primary and secondary explanatory variables, we fit a proportional hazard marginal structural model (MSM) with inverse probability of treatment weighting and inverse probability of censoring weighting for each semi-annual follow-up period, hypothesizing that time-dependent confounding variables might have been affected by past exposure to confiscation of belongings and forced displacement [28]. Figure 1 is a directed acyclic graph depicting the hypothesized relationships between the time-varying exposure (confiscation of belongings or forced displacement), the time-invariant and time-varying confounders, and the event (no longer reporting homelessness in the past 6 months during study follow-up). The proportional hazard MSM used the same covariates in the inverse probability of censoring weighting as in the inverse probability of treatment weighting as per the standard approach [28]. Event times were assumed to equal the midpoint of the last visit reporting a nonevent and the first visit reporting that an event had occurred. Visit timing was typically quite variable within each 6-month follow-up period, and occasionally visits were missed (13%), but models accounted for the number of days between visits as time-to-event models typically do. We analyzed descriptive statistics from sub-questions related to the types of housing participants were placed in, and whether they were offered housing at the time of forced displacement.

In addition, we conducted several sensitivity analyses. First, we fit a multivariable extended Cox regression model for each of the primary and secondary explanatory variables, including all potential confounders as covariates. We conducted this extended Cox regression analysis to address potentially high variance in the results of proportional hazard MSM due to relatively small sample sizes and potentially unstable weights. Second, to address the possibility of reverse causation, we lagged the primary and secondary explanatory variables to one immediately prior 6-month study follow-up cycle and re-ran the proportional hazard MSM. Third, we evaluated the potential effect modification by gender or ethnicity/ancestry by creating and adding interaction terms between gender or ethnicity/ancestry and the primary or secondary explanatory variables to the Cox regression models. Fourth, we examined the potential impacts of seasonality by adding a seasonality variable based on calendar quarters of interview dates to the Cox regression models. All *p* values were two-sided. All analyses were performed using SAS 9.4 (Cary, North Carolina, USA).

We applied an *a priori*-defined statistical protocol involving the proportional hazard MSM and Cox regression models to estimate the independent effects of the primary and secondary explanatory variables on the event. In response to the journal’s editor’s and peer reviewers’ suggestions, we have added or updated the following components to our analyses: (1) adding history of incarceration as a covariate to the analyses; (2) adding Kaplan-Meier curves; (3) adding available descriptive data on incident homelessness; (4) examining the potential effect modification by gender or ethnicity/ancestry; and (5) examining the potential impacts of seasonality.

## Results

**Fig 2:**
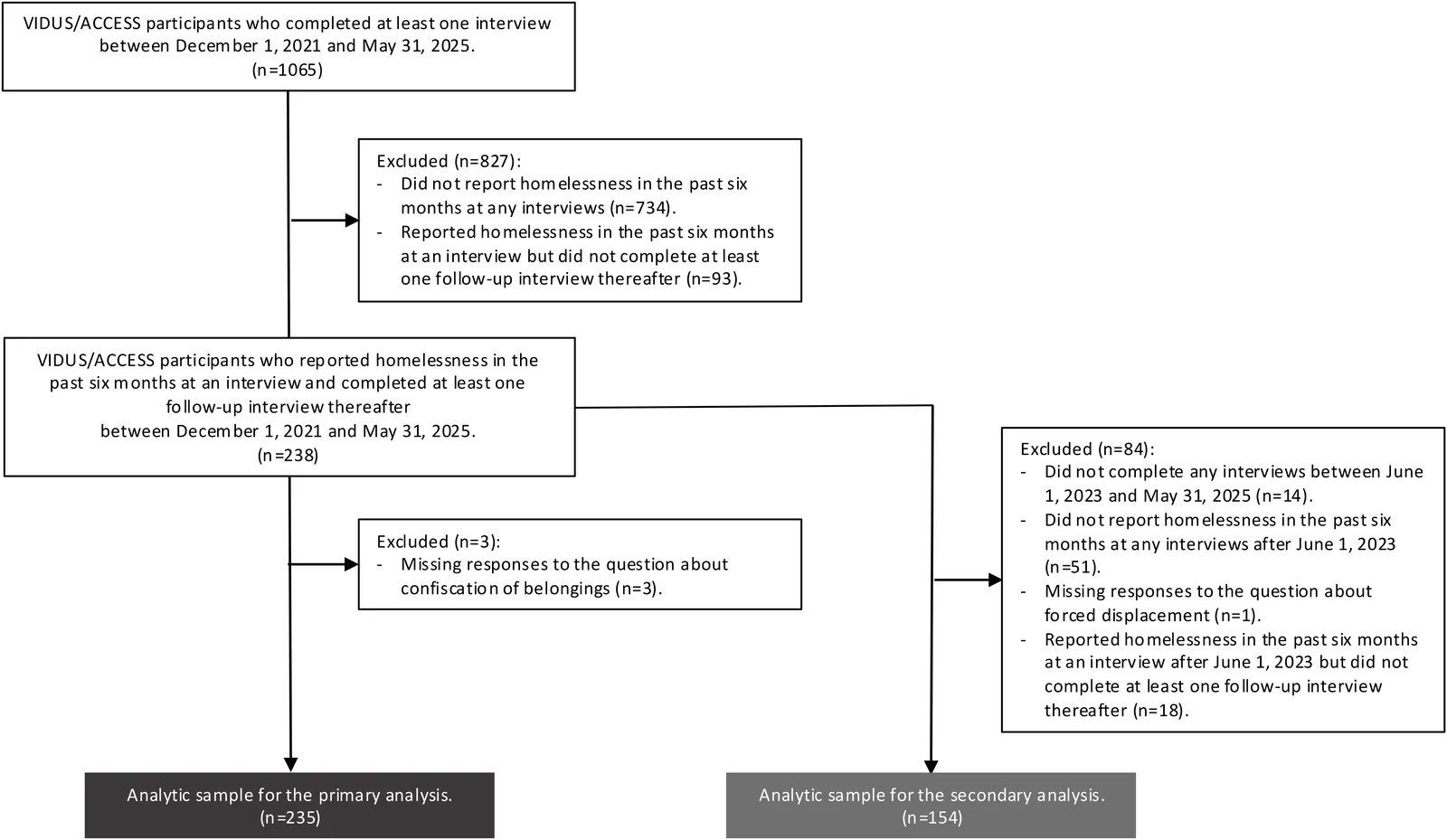
Flowchart for the determination of analytic samples. ACCESS: The AIDS Care Cohort to evaluate Exposure to Survival Services Study. VIDUS: The Vancouver Injection Drug Users Study.

**Fig 3:**
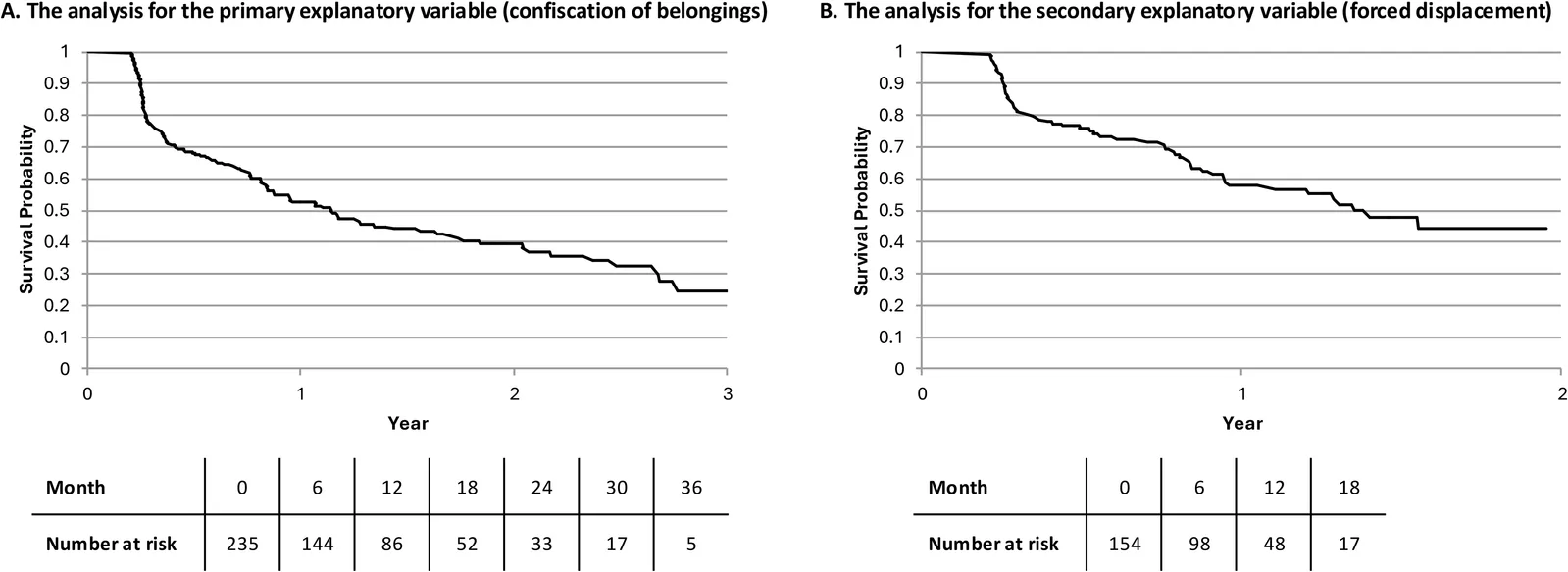
Kaplan–Meier curves for the analyses for the primary and secondary explanatory variables.

Figure 2 illustrates how we derived our analytic samples. As shown, 235 participants were eligible for the analysis of the primary explanatory variable (confiscation of belongings) and followed for 1.2 years on average (standard deviation [SD]: 0.8, interquartile range [IQR]: 0.5, 1.5), contributing 230.3 person-years of observation. A Kaplan–Meier curve and the number of participants at risk at 6-month intervals are shown in Fig 3. At baseline, the median age was 43 (interquartile range: 37–53) years, and 130 (55.3%) self-identified as men, 98 (41.7%) as women, and 7 (3.0%) as transgender or nonbinary. Furthermore, 121 (51.5%) participants self-identified as white, 103 (43.8%) as Indigenous, and 11 (4.7%) as other persons of color. The majority (*n* = 221, 94.4%) resided in the city of Vancouver in the past 6 months; of those, 190 (86.0% or 81.2% of the entire sample) lived in DTES. At baseline, 59 (25.1%) reported having their belongings confiscated in the past 6 months. Other baseline sample characteristics are presented in Table 1.

**Table 1:** Baseline sample characteristics stratified by experiencing municipal government-enforced confiscation of belongings among VIDUS/ACCESS participants who reported homelessness in the past 6 months at baseline in Vancouver, Canada, 2021−25 (*n* = 235).

Characteristic	Total *n* (%) 235 (100%)	Experienced confiscation of belongings^a^		*p* - value
		Yes *n* (*%*) 59 (25.1%)	No *n* (%) 176 (74.9%)	
**Age** (median, interquartile range)	43 (37, 53)	38 (33, 44)	45 (39, 54)	<0.001
**Self-identified ethnicity/ancestry**				0.936
White	121 (51.5%)	29 (49.2%)	92 (52.3%)	
Indigenous	103 (43.8%)	27 (45.8%)	76 (43.2%)	
Other persons of color	11 (4.7%)	3 (5.1%)	8 (4.5%)	
**Self-identified gender** ^**a**^				0.743
Man	130 (55.3%)	35 (59.3%)	95 (54.0%)	
Woman	98 (41.7%)	22 (37.3%)	76 (43.2%)	
Transgender or nonbinary	7 (3.0%)	2 (3.4%)	5 (2.8%)	
**Legally married/common law/having a regular partner**	64 (27.2%)	23 (39.0%)	41 (23.3%)	0.027
**Some or extreme barriers to mobility** ^**b**^	100 (42.6%)	29 (49.2%)	71 (40.3%)	0.287
**Moderate to severe depressive symptoms** ^**c**^	97 (48.7%)	34 (72.3%)	63 (41.4%)	<0.001
**Moderate to severe anxiety symptoms** ^**c**^	110 (55.3%)	37 (78.7%)	73 (48.0%)	<0.001
**≥ daily unregulated opioid use** ^**a,d**^	171 (72.8%)	52 (88.1%)	119 (67.6%)	0.002
**≥ daily unregulated stimulant use** ^**a,e**^	133 (56.6%)	44 (74.6%)	89 (50.6%)	0.001
**Informal or criminalized income generation activities** ^**a,f**^	146 (64.3%)	47 (82.5%)	99 (58.2%)	0.001
**Living in Downtown Eastside** ^**a,g**^	190 (81.2%)	52 (89.7%)	138 (78.4%)	0.080
**Ever incarcerated**	204 (87.2%)	54 (91.5%)	150 (85.7%)	0.273
**Enrolled in VIDUS** (vs. ACCESS)	173 (73.6%)	47 (79.7%)	126 (71.6%)	0.238

Abbreviations: ACCESS: The AIDS Care Cohort to evaluate Exposure to Survival Services Study. VIDUS: The Vancouver Injection Drug Users Study.

*P*-values were derived from the exact chi-suqare test for all variables except for age, which used the Kruskal–Wallis test.

^a^Refers to behaviors and events in the 6 months prior to an interview.

^b^Derived from EuroQol 5-Dimension 3-Level (EQ-5D-3L).

^c^Derived from Patient-Reported Outcomes Measurement Information System (PROMIS) short forms (depression 8b and anxiety 7a). Thirty-six observations out of 235 (15.3%) were missing. Percentages were calculated based on complete observations.

^d^Unregulated opioids include heroin, fentanyl or *down*.

^e^Unregulated stimulants include crystal methamphetamine, powder/crack cocaine, speedball or goofball.

^f^Eight observations out of 235 (3.4%) were missing. Percentages were calculated based on complete observations.

^g^One observation out of 235 (0.4%) were missing. Percentages were calculated based on complete observations.

During follow-up, 128 (54.5%) participants stopped reporting homelessness in the past 6 months, corresponding to an incidence rate of 55.6 (95% confidence interval [CI]: 46.4, 66.1) cases per 100 person-years. Among these 128 participants, 127 (99.2%) described the types of housing they were living in at the time of interview when they stopped reporting homelessness. The most commonly reported type of housing was SROs (*n* = 89, 70.1%), followed by apartment or house (*n* = 30, 23.6%), modular housing (*n* = 6, 4.7%), and treatment/recovery house (*n* = 1, 0.8%). One participant (0.8%) self-reported supportive housing as a type of housing that they resided in. Among the 235 participants, 111 (47.2%) individuals completed a semi-annual interview prior to the study’s baseline assessment. Among them, 87 (78.4%) individuals were identified as incident homelessness in the past 6 months at baseline. Among the 87 participants, 65 (74.7%) stopped reporting homelessness in the past 6 months during study follow-up, after a median of 0.6 year (IQR: 0.5, 1.1).

For the analysis of the secondary explanatory variable (forced displacement), the sample consisted of 154 participants, who were followed for 0.9 years on average (SD: 0.4, IQR: 0.5, 1.3), contributing 123.5 person-years. A Kaplan–Meier curve and the number of participants at risk at 6-month intervals are shown in Fig 3. During follow-up, 62 (40.3%) stopped reporting homelessness in the past 6 months, corresponding to an incidence rate of 50.2 (95% CI: 38.5, 64.3) cases per 100 person-years. Among the 154 participants, 79 (51.3%) individuals completed a semi-annual interview prior to the study’s baseline assessment. Among them, 40 (50.6%) individuals were identified as incident homelessness in the past 6 months at baseline, Among the 40 participants, 34 (85.0%) stopped reporting homelessness in the past 6 months during study follow-up, after a median of 1.0 year (IQR: 0.5, 1.7).

At baseline, 50 (32.5%) reported forced displacement in the past 6 months. During the study period, there were 121 reports/observations of forced displacement in the past 6 months. Among them, 119 (98.4%) reported that participants were not offered housing at the time of displacement, while two (1.7%) did receive an offer of housing. Among these two, one was able to successfully move into the housing offered but the other was not because “there was a line-up/no space,” which was a pre-listed response option for the question.

In the proportional hazard MSM, both confiscation of belongings and forced displacement remained negatively associated with no longer reporting homelessness in the past 6 months: adjusted hazard ratio (AHR) of 0.35 (95% CI [0.13, 0.94]; *p* = 0.036) and AHR of 0.07 (95% CI [0.02, 0.20]; *p* < 0.001), respectively. In the multivariable extended Cox regression analyses, the results were largely unchanged (see Table 2). In the sensitivity analyses where the primary and secondary explanatory variables were lagged to one immediately prior 6-month study follow-up cycle, both variables lost the statistical significance; however, the direction of the associations remained the same as in the primary analyses: AHR of 0.73 (95% CI [0.29, 1.83]; *p* = 0.497) for confiscation of belongings and AHR of 0.50 (95% CI [0.14, 1.70]; *p* = 0.264) for forced displacement. None of the interaction terms between gender or ethnicity/ancestry and the primary or secondary explanatory variables were statistically significant (all *p* > 0.3). The seasonality variable was not statistically significant (all *p* > 0.3).

**Table 2:** Bivariate and multivariable extended Cox regression analyses of securing housing among VIDUS/ACCESS participants who reported homelessness in the past 6 months at baseline in Vancouver, Canada, 2021−25.

Variable	Confiscation of belongings (*n* = 235)		Forced displacement (*n* = 154)	
	Unadjusted HR (95% CI) *p* value	Adjusted HR (95% CI) *p* value	Unadjusted HR (95% CI) *p* value	Adjusted HR (95% CI) *p* value
**Confiscation of belongings^a^**	0.19 (0.10, 0.35) *p* < 0.001	0.25 (0.12, 0.51) *p* < 0.001	NA	NA
**Forced displacement^a^**	NA	NA	0.20 (0.07, 0.55) *p* = 0.002	0.21 (0.06, 0.72) *p* = 0.012
**Age** (per year increase)	1.03 (1.01, 1.05) *p* = 0.003	1.01 (0.99, 1.03) *p* = 0.374	1.02 (1.00, 1.05) *p* = 0.070	1.00 (0.97, 1.03) *p* = 0.966
**Self-identified as white**	0.79 (0.56, 1.11) *p* = 0.172	0.91 (0.60, 1.37) *p* = 0.638	0.68 (0.41, 1.12) *p* = 0.129	0.63 (0.33, 1.20) *p* = 0.156
**Self-identified as woman, transgender or non-binary^a^**	1.22 (0.86, 1.74) *p* = 0.260	1.48 (0.95, 2.28) *p* = 0.080	1.35 (0.82, 2.22) *p* = 0.242	0.82 (0.39, 1.71) *p* = 0.591
**Legally married/common law/having a regular partner**	1.74 (1.17, 2.59) *p* = 0.006	1.55 (1.02, 2.37) *p* = 0.040	1.35 (0.73, 2.48) *p* = 0.337	1.99 (1.01, 3.89) *p* = 0.045
**Some or extreme barriers to mobility^b^**	0.92 (0.66, 1.29) *p* = 0.633	1.05 (0.72, 1.53) *p* = 0.793	0.90 (0.55, 1.48) *p* = 0.683	1.00 (0.54, 1.82) *p* = 0.991
**Moderate to severe depressive symptoms^c^**	0.57 (0.38, 0.86) *p* = 0.008	0.70 (0.43, 1.13) *p* = 0.141	0.59 (0.32, 1.09) *p* = 0.092	0.56 (0.27, 1.14) *p* = 0.110
**Moderate to severe anxiety symptoms^c^**	0.67 (0.46, 0.97) *p* = 0.034	1.02 (0.66, 1.58) *p* = 0.930	1.21 (0.70, 2.10) *p* = 0.499	1.94 (1.00, 3.77) *p* = 0.050
**≥ daily unregulated opioid use^a,d^**	0.45 (0.32, 0.63) *p* < 0.001	0.76 (0.50, 1.15) *p* = 0.196	0.48 (0.30, 0.78) *p* = 0.003	0.63 (0.37, 1.06) *p* = 0.082
**≥ daily unregulated stimulant use^a,e^**	0.78 (0.55, 1.11) *p* = 0.163	1.11 (0.72, 1.70) *p* = 0.645	0.77 (0.47, 1.27) *p* = 0.303	1.23 (0.70, 2.16) *p* = 0.467
**Informal or criminalized income generation activities^a^**	0.50 (0.35, 0.71) *p* < 0.001	0.74 (0.47, 1.17) *p* = 0.201	0.57 (0.35, 0.94) *p* = 0.029	0.90 (0.47, 1.71) *p* = 0.737
**Living in Downtown Eastside^a^**	0.45 (0.31, 0.64) *p* < 0.001	0.56 (0.36, 0.86) *p* = 0.009	0.51 (0.30, 0.84) *p* = 0.008	0.75 (0.44, 1.28) *p* = 0.296
**Ever incarcerated**	0.96 (0.57, 1.61) *p* = 0.880	1.10 (0.60, 2.04) *p* = 0.754	0.76 (0.36, 1.63) *p* = 0.484	0.60 (0.25, 1.48) *p* = 0.271

Abbreviations: ACCESS: The AIDS Care Cohort to evaluate Exposure to Survival Services Study. CI: confidence interval. HR: hazard ratio. NA: not applicable. VIDUS: The Vancouver Injection Drug Users Study.

^a^Refers to behaviors and events in the 6 months prior to an interview.

^b^Derived from EuroQol 5-Dimension 3-Level (EQ-5D-3L).

^c^Derived from Patient-Reported Outcomes Measurement Information System (PROMIS) short forms (depression 8b and anxiety 7a).

^d^Unregulated opioids include heroin, fentanyl, *down*, speedball, or goofball.

^e^Unregulated stimulants include crystal methamphetamine, powder/crack cocaine, speedball, or goofball.

## Discussion

In our cohort of unhoused people who use drugs in Vancouver, one in four reported having their belongings confiscated by city engineering workers, park rangers and/or police officers while one in three experienced municipal government-enforced forced displacement in the past 6 months at baseline. After adjusting for potential confounders, these experiences were associated with significantly lower rates of transitioning from homelessness. Among those who experienced forced displacement, the vast majority (98%) were not offered housing at the time of displacement.

Our findings corroborate previous reports based on qualitative evidence, indicating that encampment sweeps interfere with broad efforts for housing placements [8,9,11,13]. These reports suggested that interference with housing placements could occur in multiple ways, including destruction or loss of vital personal documents (e.g., identification cards) that may be required to sign a lease or join a waitlist, disrupting connections with community and services, worsening mental health, and undermining trust with public service providers, including housing service workers, as forced displacement is implemented by public workers [8,9,11,13]. Indeed, in our previous study on municipal government-enforced confiscation of belongings among unstably housed people who use drugs in the same two cohorts employed by the current study, we found that 36% of participants who had their belongings confiscated by city workers in the past 6 months reported that they tried but were unable to access housing services and 16% reported being unable to access substance use treatment in the same 6-month period [7]. These findings were mirrored in another recent cross-sectional study conducted in Massachusetts, the United States [18] and collectively demonstrate that displacement can impede access to these essential services, which in turn exacerbates the housing outcomes. Furthermore, we found that municipal government workers who forcibly removed unhoused people from public spaces almost never offered housing services to those who were displaced. These findings are consistent with the NYC Comptroller’s audit report that the number of people who were eventually housed after encampment sweeps in 2022 was only three out of 2,308 (0.1%) [23]. Taken together with previous research on the negative health impacts of street sweeps [7,12,14,15,20], our findings indicate that encampment sweeps not only increase health and safety harms, but also appear to perpetuate homelessness.

Our findings add detailed prospective evidence to recent calls for alternative policy approaches to encampment sweeps, centering on access to permanent, dignified, and affordable housing. These calls have been made by health and human rights authorities and experts in the US and Canada [9,21,29], including the American Public Health Association [8] and the Canadian Federal Housing Advocate [22], as well as those directly impacted by street sweeps in Vancouver [11]. Furthermore, amid large increases in police budgets [30], the continuation of costly forced displacement can come with an opportunity cost in the form of cuts to crucial social services and/or the acquisition or development of adequate, dignified, and affordable housing options [31].

In the province of British Columbia (B.C.), where our study setting of Vancouver is located, most encampment sweeps are carried out by municipal governments, which have legal authority over public spaces and local bylaws. The provincial government’s official stance has been to accept encampment sweeps when alternative shelter space is made reasonably available to people living in encampment [32], though it is widely known that such shelter space has never been sufficiently available [33,34]. Our findings that alternative shelter space that is reasonably available has never been offered to our study participants who were forcibly displaced demonstrate the highly problematic nature of ongoing encampment sweeps tolerated by the provincial government. Moreover, while Canada’s stock of nonmarket housing is much lower compared to the average in other countries in the Organisation for Economic Co-operation and Development (OECD) (3.5% versus 7%), Vancouver is one of the settings within Canada that suffer the most acutely from the shortages of affordable housing [35]. A report published in September 2025 estimated the need for $10.1-19.3 billion in investments to address the unmet housing needs (i.e., 29,250−54,500 affordable rental housing units) in the Metro Vancouver region over the next 5 years while only $1.2 billion was invested to create 12,500−19,500 affordable housing units in the last 5 years [35]. In addition to the severe shortages of affordable housing, since 2023 the province and the city of Vancouver have introduced several key policy changes and decisions that have limited the growth of government-subsidized, shelter-rate housing supply in Vancouver [36,37] and removed some key tenancy rights in supportive housing in B.C. [38–41], with the latter potentially increasing eviction rates. Given the ongoing encampment sweeps, our findings raise concern that these policy decisions might collectively exacerbate the structural conditions that lead to poor housing outcomes among those experiencing forced encampment sweeps.

A crucial limitation to our study is that we did not capture or assess the quality of housing that participants were placed in. In fact, the majority (70%) of participants reported ending up in SROs, which are known to have poor conditions and high rates of unlawful evictions [25,42,43]. The conditions within SROs and other government-subsidized housing have been worsening amid a dwindling supply of shelter spaces or otherwise affordable housing options across Vancouver [37]. The 2025 point-in-time homeless count report in the Greater Vancouver region also documented that 42% of respondents attributed their current state of homelessness to residential evictions [5]. Therefore, while rates of transitioning from homelessness may appear high in our study, participants continue to be at high risk of returning to homelessness after being allocated an SRO room. Future research should establish more ideal endpoints, including permanent, secure, and dignified housing.

This study has additional limitations. First, a nonrandom sample limits the generalizability of our findings. In particular, unhoused people who were the most severely impacted by encampment sweeps might have also experienced challenges in participating in our research. Therefore, the rates of transitioning from homelessness to being housed in our study might have been overestimated. Nevertheless, a cohort study of community-recruited unhoused people that ascertains exposure to and outcomes of encampment sweeps in a longitudinal manner like ours is rare. Second, self-reported data may be influenced by reporting bias, although reasonable validity of self-reports has been demonstrated in a similar drug-using population [44]. Third, as with any observational research, our findings might have been influenced by unmeasured confounding. Thus, causal inference is limited though we employed proportional hazard MSM to minimize the impacts of time-dependent confounding. In addition, we cannot exclude the possibility of reverse causation as the results of sensitivity analyses where the primary and secondary explanatory variables were lagged to one immediately prior 6-month study follow-up cycle lost the statistical significance. However, as the direction of the associations was consistent with those in the primary analyses, it might be that negative impacts of encampment sweeps on housing outcomes are more acute during the initial few months after experiencing encampment sweeps compared to at least 6 months later.

In sum, we found a high prevalence of experiencing municipal government-enforced confiscation of belongings and forced displacement among our cohort of unhoused people who use drugs in Vancouver. These experiences were associated with significantly lower rates of transitioning from homelessness. Our findings underscore the need for policy approaches that prioritize access to permanent, dignified, and affordable housing rather than practices that perpetuate homelessness.

## Supporting information

S1 Checklist STROBE Checklist. (DOCX)

S1 Data Minimal dataset. (PDF)

## Abbreviations

ACCESS: AIDS Care Cohort to evaluate Exposure to Survival Services
AHR: adjusted hazard ratio
CI: confidence interval
CREW: Community Research Ethics Workshop
HIV: Human Immunodeficiency Virus
IQR: interquartile range
MSM: marginal structural modeling
NYC: New York City
PROMIS: Patient-Reported Outcomes Measurement Information System
SD: standard deviation
SROs: single-room occupancy hotels
US: United States
VIDUS: Vancouver Injection Drug Users Study
