# Supplementary material for: Encampment sweeps and persistent homelessness among unhoused people who use drugs in Vancouver, Canada: A prospective cohort study
Source: PLoS Med. 2026 Sep 24;23(9):e1005033. doi: 10.1371/journal.pmed.1005033 (PMC13606962; doi:10.1371/journal.pmed.1005033)
Supplement: Supplementary file 1 [file pmed.1005033.s002.pdf]

S2: Minimal dataset

The values behind the means, standard deviations and other measures reported among the sample of n=235

	n	mean	standard deviation	minimum	lower quartile	median	upper quartile	maximum
Follow-up years	235	1.155501187	0.757772218	0.399726215	0.517453799	0.966461328	1.516769336	3.34017796
Age	235	44.66971325	10.21027456	26.64476386	36.57494867	42.72416153	52.62149213	74.15742642

Baseline age stratified by confiscation of belongings in the past six months

	KruskalWallis P value	confiscation of belongings	n	mean	standard deviation	minimum	lower quartile	median	upper quartile	maximum
Age	<0.001	No	176	46.50418456	10.21451747	27.73716632	38.72689938	44.93086927	53.89596167	74.15742642
		Yes	59	39.19739208	8.069146533	26.64476386	32.93086927	37.98767967	44.16427105	58.76796715

The values behind the means, standard deviations and other measures reported among the sample of n=65

	n	mean	standard deviation	minimum	lower quartile	median	upper quartile	maximum
Years of follow-up before stopping reporting homelessness	65	0.893845101	0.582985072	0.413415469	0.495550992	0.59137577	1.100616016	2.973305955

The values behind the means, standard deviations and other measures reported among the sample of n=154

	n	mean	standard deviation	minimum	lower quartile	median	upper quartile	maximum
Follow-up years	154	0.925944692	0.439280228	0.399726215	0.517453799	0.815879535	1.281314168	1.952087611
Age	154	44.7682516	10.20511318	26.64476386	36.54209446	43.4770705	53.45379877	74.15742642

The values behind the means, standard deviations and other measures reported among the sample of n=34

	n	mean	standard deviation	minimum	lower quartile	median	upper quartile	maximum
Years of follow-up before stopping reporting homelessness	34	1.221000926	0.77982127	0.429842574	0.542094456	0.948665298	1.73853525	2.973305955

The values behind the Kaplan-Meier curve for the analysis for the primary explanatory variable (confiscation of belongings)

Year	Survival Probability
0	1
0.202600958	0.995744681
0.205338809	0.991489362
0.206707734	0.987234043
0.210814511	0.982978723
0.212183436	0.978723404
0.213552361	0.974468085
0.214921287	0.970212766
0.219028063	0.965957447
0.220396988	0.961702128
0.221765914	0.953191489
0.224503765	0.94893617
0.22587269	0.944680851
0.228610541	0.940425532
0.234086242	0.936170213
0.235455168	0.931914894
0.236824093	0.927659574
0.238193018	0.919148936
0.240930869	0.914893617
0.242299795	0.910638298
0.245037645	0.89787234
0.246406571	0.893617021
0.247775496	0.885106383
0.250513347	0.872340426
0.251882272	0.863829787
0.254620123	0.859574468
0.255989049	0.855319149
0.257357974	0.842553191
0.258726899	0.838297872
0.260095825	0.825531915
0.265571526	0.817021277
0.268309377	0.808510638
0.269678303	0.8
0.271047228	0.795744681
0.273785079	0.791489362
0.277891855	0.782978723

Year	Survival Probability
0.283367556	0.778723404
0.288843258	0.774468085
0.295687885	0.770212766
0.299794661	0.765957447
0.313483915	0.757446809
0.328542094	0.753191489
0.343600274	0.74893617
0.346338125	0.744680851
0.34770705	0.740425532
0.354551677	0.736170213
0.355920602	0.731914894
0.362765229	0.727659574
0.36550308	0.719148936
0.366872005	0.714893617
0.373716632	0.710638298
0.395619439	0.706382979
0.399726215	0.706382979
0.405201916	0.706382979
0.407939767	0.702075765
0.414784394	0.697768552
0.428473648	0.693461339
0.438056126	0.693461339
0.443531828	0.693461339
0.446269678	0.693461339
0.45174538	0.689044388
0.458590007	0.684627436
0.473648186	0.684627436
0.476386037	0.684627436
0.479123888	0.684627436
0.492813142	0.684627436
0.494182067	0.680001575
0.495550992	0.680001575
0.498288843	0.680001575
0.501026694	0.680001575
0.503764545	0.67524632
0.506502396	0.67524632
0.514715948	0.67524632
0.517453799	0.67524632
0.52019165	0.67524632
0.5229295	0.67524632

Year	Survival Probability
0.529774127	0.670281273
0.533880903	0.670281273
0.542094456	0.670281273
0.550308008	0.670281273
0.55578371	0.665164622
0.561259411	0.665164622
0.581793292	0.659968024
0.59137577	0.659968024
0.594113621	0.659968024
0.602327173	0.654688279
0.6091718	0.649408535
0.629705681	0.649408535
0.64476386	0.644085514
0.66255989	0.644085514
0.681724846	0.638718135
0.709103354	0.633350756
0.711841205	0.633350756
0.720054757	0.627937502
0.741957563	0.622524247
0.758384668	0.617110993
0.761122519	0.611697739
0.76659822	0.606284484
0.767967146	0.600822462
0.769336071	0.600822462
0.804928131	0.600822462
0.807665982	0.600822462
0.809034908	0.595207299
0.810403833	0.589592135
0.824093087	0.583976972
0.83504449	0.578361809
0.839151266	0.572746646
0.843258042	0.567131483
0.845995893	0.56151632
0.87063655	0.56151632
0.878850103	0.550172555
0.881587953	0.550172555
0.884325804	0.550172555
0.93908282	0.550172555
0.948665298	0.538466756
0.950034223	0.532613857

Year	Survival Probability
0.960985626	0.526760957
0.966461328	0.526760957
0.982888433	0.526760957
0.991101985	0.526760957
0.996577687	0.526760957
1.015742642	0.526760957
1.032169747	0.526760957
1.037645448	0.526760957
1.045859001	0.526760957
1.054072553	0.526760957
1.062286105	0.526760957
1.063655031	0.520176445
1.066392882	0.513591933
1.092402464	0.513591933
1.104722793	0.506921908
1.138945927	0.506921908
1.140314853	0.50016295
1.14715948	0.493403991
1.152635181	0.493403991
1.155373032	0.493403991
1.163586585	0.493403991
1.170431211	0.486355362
1.173169062	0.479306734
1.174537988	0.472258105
1.199178645	0.472258105
1.212867899	0.472258105
1.215605749	0.472258105
1.25119781	0.472258105
1.277207392	0.464641039
1.281314168	0.457023973
1.319644079	0.457023973
1.338809035	0.457023973
1.348391513	0.449144249
1.399041752	0.449144249
1.434633812	0.441123816
1.445585216	0.441123816
1.456536619	0.441123816
1.478439425	0.441123816
1.533196441	0.441123816
1.557837098	0.43247433

Year	Survival Probability
1.568788501	0.43247433
1.612594114	0.43247433
1.626283368	0.43247433
1.641341547	0.423272748
1.664613279	0.423272748
1.734428474	0.413866687
1.760438056	0.404460626
1.828884326	0.404460626
1.837097878	0.404460626
1.838466804	0.394595733
1.861738535	0.394595733
1.894592745	0.394595733
1.900068446	0.394595733
1.94934976	0.394595733
1.952087611	0.394595733
1.954825462	0.394595733
1.993155373	0.394595733
2.034223135	0.394595733
2.039698836	0.382264616
2.07118412	0.369933499
2.099931554	0.369933499
2.130047912	0.369933499
2.165639973	0.369933499
2.176591376	0.356232259
2.275154004	0.356232259
2.321697467	0.356232259
2.372347707	0.341389248
2.406570842	0.341389248
2.414784394	0.341389248
2.439425051	0.341389248
2.483230664	0.324319786
2.494182067	0.324319786
2.524298426	0.324319786
2.546201232	0.324319786
2.568104038	0.324319786
2.64202601	0.324319786
2.67761807	0.29937211
2.680355921	0.274424434
2.685831622	0.274424434
2.737850787	0.274424434

Year	Survival Probability
2.767967146	0.24393283
3.34017796	0.24393283

The values behind the Kaplan-Meier curves for the analysis for the secondary explanatory variable (forced displacement)

Year	Survival Probability
0	1
0.213552361	0.993506494
0.214921287	0.987012987
0.219028063	0.974025974
0.224503765	0.967532468
0.22587269	0.961038961
0.231348392	0.954545455
0.232717317	0.941558442
0.242299795	0.935064935
0.249144422	0.928571429
0.250513347	0.915584416
0.251882272	0.902597403
0.255989049	0.896103896
0.258726899	0.88961039
0.260095825	0.87012987
0.266940452	0.863636364
0.268309377	0.857142857
0.271047228	0.850649351
0.277891855	0.844155844
0.283367556	0.837662338
0.288843258	0.824675325
0.295687885	0.818181818
0.29705681	0.811688312
0.313483915	0.805194805
0.346338125	0.798701299
0.353182752	0.792207792
0.366872005	0.785714286
0.392881588	0.779220779
0.399726215	0.779220779
0.405201916	0.779220779
0.407939767	0.772617213
0.438056126	0.772617213
0.442162902	0.765839869
0.443531828	0.765839869
0.446269678	0.765839869
0.45174538	0.765839869
0.473648186	0.765839869
0.476386037	0.765839869
0.479123888	0.765839869
0.492813142	0.765839869
0.494182067	0.758404531
0.495550992	0.758404531
0.498288843	0.758404531
0.514715948	0.758404531
0.517453799	0.758404531
0.52019165	0.758404531
0.528405202	0.750249643
0.533880903	0.750249643
0.535249829	0.741913536
0.542094456	0.741913536
0.550308008	0.741913536
0.55578371	0.733385794
0.558521561	0.733385794
0.561259411	0.733385794
0.572210815	0.733385794
0.594113621	0.733385794
0.6091718	0.724331649
0.635181383	0.724331649
0.640657084	0.724331649
0.66255989	0.724331649
0.703627652	0.714924744
0.711841205	0.714924744
0.728268309	0.714924744
0.736481862	0.714924744
0.758384668	0.705131255
0.762491444	0.695337765
0.76659822	0.695337765
0.769336071	0.695337765
0.787132101	0.685260406
0.792607803	0.675183047
0.804928131	0.675183047
0.806297057	0.664953001
0.815879535	0.664953001
0.83504449	0.65456311
0.839151266	0.64417322
0.843258042	0.633783329

Year	Survival Probability
0.87063655	0.633783329
0.878850103	0.623220274
0.881587953	0.623220274
0.892539357	0.623220274
0.907597536	0.612286585
0.93908282	0.612286585
0.950034223	0.590021618
0.960985626	0.578889134
0.966461328	0.578889134
0.977412731	0.578889134
0.982888433	0.578889134
0.991101985	0.578889134
1.032169747	0.578889134
1.037645448	0.578889134
1.045859001	0.578889134
1.048596851	0.578889134
1.104722793	0.565732563
1.11156742	0.565732563
1.138945927	0.565732563
1.152635181	0.565732563
1.185489391	0.565732563
1.199178645	0.565732563
1.210130048	0.550844864
1.215605749	0.550844864
1.25119781	0.550844864
1.259411362	0.550844864
1.281314168	0.550844864
1.289527721	0.534152596
1.305954825	0.517460327
1.319644079	0.517460327
1.32238193	0.517460327
1.338809035	0.517460327
1.344284736	0.517460327
1.353867214	0.49829513
1.379876797	0.49829513
1.399041752	0.478363325
1.420944559	0.478363325
1.434633812	0.478363325
1.456536619	0.478363325
1.45927447	0.478363325

Year	Survival Probability
1.46201232	0.478363325
1.475701574	0.478363325
1.516769336	0.478363325
1.533196441	0.478363325
1.549623546	0.478363325
1.552361396	0.444194516
1.952087611	0.444194516
